# MITOCHONDRIAL METABOLIC CHECKPOINT, STEM CELL AGING AND REJUVENATION

**DOI:** 10.1093/geroni/igac059.1143

**Published:** 2022-12-20

**Authors:** Danica Chen

**Affiliations:** University of California - Berkeley, Berkeley, California, United States

## Abstract

Cell cycle checkpoints are surveillance mechanisms in eukaryotic cells that monitor the condition of the cell, repair cellular damages, and allow the cell to progress through the various phases of the cell cycle when conditions become favorable. Recent advances in stem cell biology highlight a mitochondrial metabolic checkpoint that is essential for stem cells to return to the quiescent state. As quiescent stem cells enter the cell cycle, mitochondrial biogenesis is induced and mitochondrial stress is increased. Mitochondrial unfolded protein response and mitochondrial oxidative stress response are activated to alleviate stresses and allow stem cells to exit the cell cycle and return to quiescence. These processes are critically regulated by several sirtuin family members, which are NAD+-dependent deacetylases. Because loss of stem cell quiescence results in the depletion of the stem cell pool and compromised tissue regeneration, deciphering the molecular mechanisms that regulate the mitochondrial metabolic checkpoint in stem cells will increase our understanding of tissue homeostasis and how it becomes dysregulated under pathological conditions and during aging. More broadly, this knowledge is instrumental for understanding the maintenance of cells that convert between quiescence and proliferation to support their physiological functions.

